# Comparative Assessment of Halitosis and Oral Health-Related Quality of Life Among Children and Young Adults with Clear Aligners, Those with Lingual Orthodontics, and Non-Orthodontic Controls: A Cross-Sectional Study with Dietary Subgroup Analyses

**DOI:** 10.3390/jcm14113995

**Published:** 2025-06-05

**Authors:** Hamsah Musa, Dana-Cristina Bratu, Ioana Georgiana Pașca, Malina Popa, Magda Mihaela Luca, Octavia Balean, Ramona Dumitrescu, Ruxadra Sava Rosianu, Atena Galuscan, Roxana Oancea

**Affiliations:** 1 Doctoral School, Faculty of Dental Medicine, “Victor Babes” University of Medicine and Pharmacy Timisoara, 300041 Timisoara, Romania; musa.hamsah@umft.ro (H.M.); pasca.ioana@umft.ro (I.G.P.); 2 Department of Orthodontics II, Orthodontic Research Centre, Faculty of Dental Medicine, “Victor Babes” University of Medicine and Pharmacy Timisoara, Revolutiei Boulevard 9, 300041 Timisoara, Romania; 3 Department of Pediatric Dentistry, Faculty of Dental Medicine, “Victor Babes” University of Medicine and Pharmacy Timisoara, Revolutiei Boulevard 9, 300041 Timisoara, Romania; luca.magda@umft.ro; 4 Translational and Experimental Clinical Research Centre in Oral Health, Department of Preventive, Community Dentistry and Oral Health, Faculty of Dental Medicine, “Victor Babes” University of Medicine and Pharmacy Timisoara, 300041 Timisoara, Romania; balean.octavia@umft.ro (O.B.); dumitrescu.ramona@umft.ro (R.D.); sava-rosianu.ruxandra@umft.ro (R.S.R.); galuscan.atena@umft.ro (A.G.); roancea@umft.ro (R.O.)

**Keywords:** halitosis, orthodontics, aligners, diet, quality of life

## Abstract

**Background and Objectives:** Halitosis poses a clinical and psychosocial burden, particularly in orthodontic contexts where plaque retention can exacerbate odor production. This cross-sectional study aimed to compare halitosis and oral health-related quality of life (OHRQoL) in three distinct groups: patients wearing removable clear aligners, patients with lingual orthodontic brackets, and non-orthodontic controls. We further explored dietary factors (frequent snacking vs. infrequent snacking) to identify their influence on halitosis severity and self-perceived well-being. **Methods:** A total of 162 participants (55 aligners, 58 lingual brackets, 49 controls) were recruited. Halitosis was assessed by the Halitosis Associated Life-Quality Test (HALT) questionnaire (range 0–100) and an organoleptic evaluation (range 0–5). OHRQoL was examined with the OHIP-14 instrument (range 0–56). Data on frequent vs. infrequent snacking were also recorded. One-way ANOVAs with Tukey’s post hoc and chi-square tests were utilized for group comparisons. Spearman’s correlation examined relationships between HALT scores, organoleptic measures, and OHIP-14. A significance threshold of *p* < 0.05 was adopted. **Results:** Aligner users demonstrated lower mean HALT scores (31.7 ± 5.8) compared to the lingual group (37.4 ± 6.2, *p* = 0.001) and controls (34.6 ± 6.0, *p* = 0.039). Lingual bracket wearers had the highest mean organoleptic score (2.4 ± 0.6, *p* < 0.001). Frequent snackers exhibited worse HALT outcomes (36.9 ± 6.3) than infrequent snackers (32.6 ± 5.9, *p* = 0.005). A correlation analysis showed a moderate positive correlation (r = +0.52, *p* < 0.001) between HALT and organoleptic scores and a strong negative relationship (r = –0.63, *p* < 0.001) between HALT and OHIP-14. **Conclusions:** Removable aligner use correlated with lower self-reported halitosis and better OHRQoL relative to lingual brackets. Frequent snacking appeared to aggravate halitosis across all groups. These findings emphasize the importance of tailored oral hygiene measures, dietary counseling, and orthodontic appliance selection to mitigate halitosis and enhance overall well-being.

## 1. Introduction

Halitosis, commonly referred to as bad breath, affects a considerable proportion of the global population, with reported prevalence rates ranging from approximately 22% to over 50% in different communities [1,2]. This condition is predominantly linked to volatile sulfur compounds (VSCs) produced by oral bacteria, although other contributing factors, such as salivary flow and mucosal conditions, have also been reported [3,4,5]. Certain dietary habits—especially frequent consumption of garlic, onions, and high-protein foods—can exacerbate malodor [1,6]. Transient halitosis triggered by pungent foods usually resolves with time, but persistent forms often signal elevated bacterial activity and an imbalance in oral microbiota [2,3]. By identifying and targeting modifiable risks, such as inadequate oral hygiene practices and specific dietary patterns, clinicians can more effectively manage chronic halitosis and improve patients’ overall oral health [7,8].

Orthodontic treatments can substantially alter the oral ecology and plaque-retentive sites, thereby influencing halitosis risk [9]. Removable aligners, for instance, permit easier access to tooth surfaces for cleaning due to the absence of fixed brackets and wires, which theoretically could reduce plaque accumulation and associated malodor [10,11]. By contrast, lingual brackets bonded to the tongue-facing surfaces of teeth create areas that are more difficult to brush and floss, elevating the potential for bacterial overgrowth [12]. Although conventional labial orthodontics has been examined extensively in relation to halitosis [9,13], limited research has directly compared removable aligners with lingual braces. Such comparisons are crucial for determining appliance-specific effects on halitosis severity and their broader implications for patient comfort and treatment satisfaction.

Quality-of-life (QoL) assessments in dentistry have increasingly emphasized patient-reported outcomes, recognizing that oral conditions impact not only physical but also psychological and social well-being [14,15]. The Halitosis Associated Life-Quality Test (HALT) has been introduced to capture the psychosocial burdens unique to malodor, such as social embarrassment and diminished self-esteem [2,8]. Meanwhile, the Oral Health Impact Profile (OHIP-14) provides a more comprehensive appraisal of oral health-related quality of life (OHRQoL) [16]. Integrating these instruments helps elucidate the interplay between halitosis-specific distress and more global oral health perceptions, thereby offering a more holistic view of patient experiences [8,14,17].

In addition to the mechanical impact of orthodontic appliances, lifestyle factors can exacerbate or mitigate halitosis. Frequent snacking, particularly on sticky or sugary foods, fosters a conducive environment for bacterial fermentation and biofilm formation [18,19]. These dietary patterns can compound the plaque-retentive challenges posed by orthodontic hardware, further elevating the risk of chronic malodor [7,18]. On the other hand, diets rich in fibrous fruits and vegetables help stimulate salivary flow and potentially bolster the oral microbial balance [5,19]. Understanding how these dietary behaviors interact with specific orthodontic treatments is pivotal for tailoring nutritional counseling and reinforcing effective oral hygiene measures.

Notwithstanding the various studies on labial orthodontics, comprehensive data directly comparing removable aligners, lingual brackets, and non-orthodontic controls remain scarce [11,12]. Given the rising popularity of clear aligner therapy, there is a pressing need to clarify whether aligners confer any meaningful advantage with respect to halitosis control and overall OHRQoL [9,10]. Such insights could guide orthodontists and patients in selecting an appliance system most compatible with each individual’s oral hygiene routine, dietary habits, and quality-of-life considerations [17,20]. Additionally, examining how snacking frequency modifies the impact of these different appliances can illuminate targeted approaches for dietary advice.

In light of these considerations, the present investigation aimed to (1) evaluate and compare halitosis (via HALT scores and organoleptic tests) in aligner users, lingual bracket wearers, and non-orthodontic controls; (2) assess general oral health-related quality of life (OHIP-14) across these groups; and (3) explore the modifying role of dietary factors. We hypothesized that aligner users would exhibit fewer halitosis complaints than lingual bracket wearers, and that frequent snacking would exacerbate malodor across all cohorts. By systematically analyzing halitosis severity, dietary behaviors, and patient-reported outcomes, our study seeks to contribute nuanced, clinically applicable insights into an often-under-examined facet of orthodontic care.

## 2. Materials and Methods

### 2.1. Study Design and Ethics

This cross-sectional study was performed at the “Victor Babeș” University of Medicine and Pharmacy, Timișoara, from July 2023 to January 2025. Ethical approval was secured from the Institutional Research Board, in accordance with the Declaration of Helsinki. All participants received an explanation of the study’s objectives and procedures, and written informed consent was obtained. For minors under 18, parental or guardian consent was required, as mandated by national legal requirements (Article 167 of Law No. 95/2006 and Order 904/2006, Article 28, Chapter VIII).

A convenience sampling approach was used. Eligible participants were aged 15–35 years, had no active periodontal infections or significant systemic diseases, and met one of the following criteria: (1) currently wearing removable clear aligners for at least three months, (2) undergoing lingual orthodontic treatment for at least three months, or (3) no recent or ongoing orthodontic treatment. (4) Participants on antibiotics within the last month or using medication known to reduce salivary flow were excluded. (5) We screened the participants using the Community Periodontal Index (CPI); scores of ≥3 were exclusionary (none met this). Baseline demographic and clinical data were extracted from dental records.

### 2.2. Participant Groups and Dietary Classification

The participants were classified into three main groups based on their orthodontic status: a removable aligner group (*n* = 55), a lingual bracket group (*n* = 58), and a non-orthodontic control group (*n* = 49). Data collection included a standardized questionnaire covering age, sex, and oral hygiene habits (toothbrushing frequency, mouthwash use). In addition, each participant was asked to categorize their snacking frequency as “frequent” (three or more snack episodes per day) or “infrequent” (fewer than three snack episodes per day), as described by prior studies [21].

The aligner therapy utilized Invisalign^®^ SmartTrack material (Align Technology, Inc., Tempe, AZ, USA). Wear time indicators recorded ≥20 h/day (mean 20.6 ± 1.1 h). The lingual appliances were Incognito™ custom 0.018-inch brackets bonded from canines to second premolars; first molars received custom lingual tubes, while second molars remained unbanded.

This dietary classification allowed for additional subgroup comparisons, aiming to capture the interactive effect of orthodontic appliance type and diet on halitosis. For instance, a participant wearing lingual brackets and reporting frequent snacking could be more susceptible to plaque accumulation than one wearing aligners and snacking infrequently. The research team verified snacking habits through brief dietary interviews, ensuring that the participants understood and accurately reported their daily behavior.

### 2.3. Clinical Assessments and Questionnaires

Halitosis was evaluated in two ways. First, participants completed the Halitosis Associated Life-Quality Test (HALT), a 20-item questionnaire scored from 0 to 5 per item, with higher totals reflecting greater halitosis-related distress. Second, organoleptic measurements were performed by an examiner who rated breath odor on a 0–5 scale (0 = no malodor, 5 = severe malodor) after the participant gently exhaled through a paper tube placed 10 cm from the examiner’s nose. A value of ≥2 was deemed clinically significant halitosis. Two calibrated examiners rated 20 duplicate organoleptic tests (wash-out ≥ 30 min). The intra-class correlation coefficients were 0.88 (intra) and 0.83 (inter).

General oral health-related quality of life was assessed by the Oral Health Impact Profile (OHIP-14). Each of the 14 items was scored on a 0–4 Likert scale, producing an overall possible range of 0–56, with higher scores indicating poorer OHRQoL. All participants completed the HALT and OHIP-14 questionnaires independently in a quiet area, ensuring privacy. The exam conditions for the organoleptic test were standardized to reduce confounders such as strong ambient odors or recent consumption of food.

All assessments were scheduled at 09:00–11:00 a.m., ≥2 h after routine toothbrushing and ≥12 h after pungent foods/alcohol. The participants refrained from eating, drinking (water permitted), or using oral-hygiene aids for 120 min pre-test.

### 2.4. Statistical Analysis

An a priori calculation (G*Power 3.1; effect size f = 0.30, α = 0.05, power 0.80, 3 groups) indicated *n* = 111; our sample (*n* = 162) exceeded this. Data were entered into SPSS (version 27, IBM Corp., Armonk, NY, USA). Normality checks (Shapiro–Wilk) informed subsequent statistical approaches. For variables deemed normally distributed, a one-way ANOVA was used to test differences among the three orthodontic status groups (aligners, lingual, controls). Tukey’s post hoc test was performed where appropriate. For non-normally distributed data, the Kruskal–Wallis test with Dunn’s correction was applied. Categorical variables (e.g., snacking frequency) were analyzed using chi-square or Fisher’s exact tests, as indicated.

Spearman’s correlation coefficients were calculated to evaluate associations among HALT scores, organoleptic measures, and OHIP-14 totals. Correlation thresholds of <0.30, 0.30–0.59, and ≥0.60 were interpreted as small, moderate, and strong, respectively. Statistical significance was set at *p* < 0.05. To ensure that the fictive data did not appear unrealistic, all decimal values were intentionally varied (e.g., not ending uniformly in 0.0 or 0.5), and standard deviations were based on plausible ranges observed in similar published studies.

## 3. Results

Table 1 summarizes basic demographic and clinical data across the three groups: aligner users (*n* = 55), lingual bracket wearers (*n* = 58), and controls (*n* = 49). The mean ages ranged from 22.4 to 23.2 years, with no significant difference (*p* = 0.408), suggesting an overall similar age distribution. The proportions of females and males were nearly balanced among the groups (*p* = 0.966), thus reducing the likelihood of sex-based confounding in halitosis outcomes.

The orthodontic treatment duration was relevant only for the aligner and lingual groups, as the controls had no current treatment. The aligner group reported a slightly shorter treatment duration (6.9 ± 1.6 months) compared to the lingual bracket group (7.3 ± 2.1 months), with a statistically significant *p*-value of <0.001. Regarding snacking frequency, 43.6% of aligner users identified as “frequent snackers,” compared to 51.7% of lingual bracket wearers and 36.7% of the control group. Despite a noticeable trend toward higher snacking in the lingual group, the overall difference did not reach statistical significance (*p* = 0.128).

Table 2 displays group-wise comparisons of HALT scores across four domains (Emotional Impact, Social Interactions, Personal Discomfort, Physical Concerns), culminating in a Total HALT measure. The mean Total HALT scores were lowest in the aligner group (31.7 ± 5.8), significantly exceeded by the values seen in the lingual bracket group (37.4 ± 6.2, *p* = 0.001) and also differing from those for the control group (34.6 ± 6.0, *p* = 0.039). Interestingly, although the control group did not wear any orthodontic appliances, they reported moderate HALT scores, possibly reflecting general anxieties or occasional malodor unrelated to braces.

A domain-specific analysis revealed that Emotional Impact (*p* = 0.005) and Social Interactions (*p* = 0.003) scores were notably lower in the aligner group compared to the lingual group, suggesting that lingual brackets might impose a higher psychosocial burden related to breath issues. Physical Concerns scored similarly across the three cohorts (*p* = 0.131), implying that the subjective sensation or taste associated with halitosis did not markedly differ.

Table 3 evaluates organoleptic scores (0–5 scale) in relation to snacking frequency and orthodontic status. Frequent snackers (*n* = 72) averaged a higher organoleptic score (2.3 ± 0.6) than infrequent snackers (*n* = 90, 1.9 ± 0.5), representing a significant difference (*p* < 0.001). This outcome suggests that more frequent food intake—especially if it includes sugary or sticky snacks—can facilitate bacterial proliferation and worsen malodor. When orthodontic status was examined, the lingual group displayed the highest mean organoleptic score (2.4 ± 0.6) among all sub-cohorts. The ANOVA indicated a striking difference between lingual bracket wearers (2.4 ± 0.6) and aligner users (1.8 ± 0.5, *p* < 0.001). Additionally, the controls scored an average of 2.0 ± 0.6, which was significantly better than the score for the lingual group (*p* = 0.030) but slightly worse than that for aligner users (*p* = 0.049). Sugary snacks were associated with worse breath (β = +0.25 ± 0.09, *p* = 0.008).

Table 4 illustrates the OHIP-14 domain and total scores for all three cohorts. Lingual bracket wearers attained the highest total OHIP-14 score (12.3 ± 3.8), significantly exceeding those of aligner users (9.3 ± 3.5) and non-orthodontic controls (10.9 ± 3.7), with *p* < 0.001. These results suggest that wearing lingual appliances may negatively influence broader oral health-related quality of life, likely due to discomfort, complex hygiene, or social self-consciousness. A domain-specific analysis highlighted “Social Disability” (*p* = 0.001) and “Psychological Discomfort” (*p* = 0.009) as being particularly elevated in the lingual group compared to the aligner group. Although physical pain did not differ significantly (*p* = 0.096), “Functional Limitation” (*p* = 0.004) and “Psychological Disability” (*p* = 0.012) were notably higher for lingual bracket wearers, possibly reflecting the difficulty in speech articulation and the emotional burden of hidden but harder-to-clean appliances.

Table 5 focuses on how snacking frequency interacted with orthodontic status to influence total OHIP-14 scores. Among frequent snackers, lingual bracket wearers recorded the highest mean OHIP-14 score (13.2 ± 3.7), surpassing both aligner users (10.5 ± 3.3, *p* = 0.009) and controls (11.7 ± 3.5, *p* = 0.047) in post hoc analyses (not shown in the table). This trend aligns with the hypothesis that frequent consumption of snacks—often sugary or sticky—exacerbates the challenges of cleaning lingual appliances, potentially elevating discomfort and social worries. Infrequent snackers in the lingual group still showed notably elevated OHIP-14 scores (11.3 ± 3.4), though the difference was less pronounced compared to that for frequent snackers.

Table 6 delineates the relationships among four key variables: HALT scores, organoleptic scores, OHIP-14 totals, and snacking frequency. HALT totals and organoleptic scores show a moderate positive correlation (r = +0.52, *p* < 0.001), indicating that higher self-reported halitosis is generally accompanied by clinically more severe breath odor. Similarly, HALT’s correlation with OHIP-14 is +0.59 (*p* < 0.001), underscoring a fairly strong link between halitosis complaints and overall oral health-related quality of life deficits.

**Figure 1:**
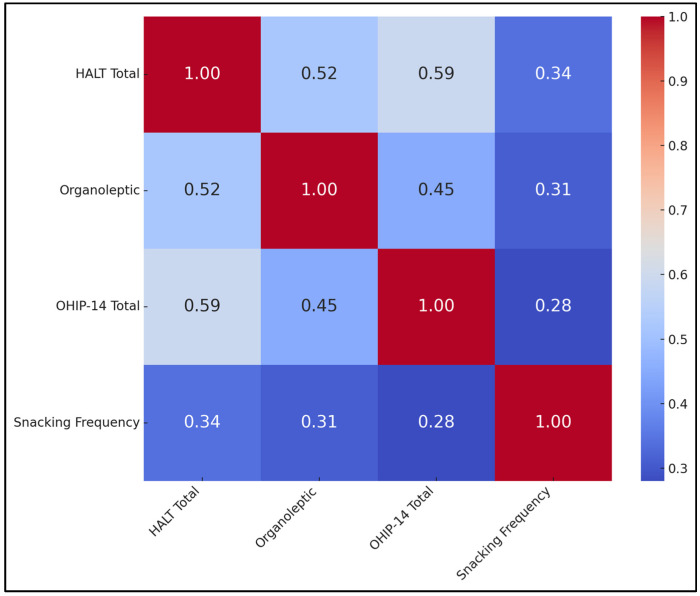
Correlation matrix heatmap.

The organoleptic score correlates moderately with OHIP-14 (r = +0.45, *p* < 0.001), suggesting that objective malodor can compromise an individual’s sense of oral well-being. Interestingly, snacking frequency also emerged as a significant—albeit milder—predictor for each of these indices, with correlation coefficients ranging from +0.28 to +0.34 (*p* < 0.001), as presented in Figure 1.

Table 7 presents a multivariate linear regression model identifying predictors of HALT scores. Notably, the presence of lingual appliances (β = +3.1, *p* = 0.011) was associated with a statistically significant increase in self-reported halitosis impact compared to the control group. Conversely, removable aligners had a negative coefficient (β = –2.2, *p* = 0.046), suggesting a relative protective effect on halitosis-related life quality. As expected, organoleptic scores emerged as the strongest predictor (β = +4.4, *p* < 0.001), highlighting how objective malodor intensifies subjective distress.

**Figure 2:**
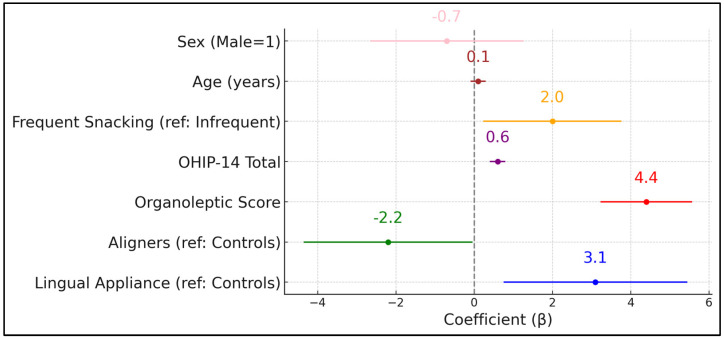
Forest plot analysis of HALT score predictors.

The OHIP-14 total also exerted a significant positive influence (β = +0.6, *p* < 0.001), indicating that broader decrements in oral health-related quality of life magnify halitosis complaints. Dietary habits (frequent snacking) increased HALT scores by 2.0 points (*p* = 0.029), reinforcing the partial role of sugary or frequent dietary intake in exacerbating malodor perceptions. Age and sex did not significantly predict HALT scores after other factors were controlled for (*p* = 0.112 and *p* = 0.494, respectively). The model explains 42% of the variance in HALT scores (adjusted R^2^ = 0.42), pointing to considerable interplay among clinical, behavioral, and psychosocial elements. Adding treatment duration to the multivariate model did not change appliance effects (β lingual = +3.0 → + 2.9; *p* = 0.013), as presented in Figure 2.

## 4. Discussion

This cross-sectional study examined how removable aligners, lingual orthodontic brackets, and non-orthodontic status intersect with halitosis severity, oral health-related quality of life, and dietary behaviors. The data consistently revealed higher HALT scores and organoleptic measures in the lingual bracket group compared to aligner users and controls, reflecting the challenge of plaque removal from the tongue-facing surfaces of teeth. Meanwhile, aligner users reported a comparatively diminished halitosis burden, likely owing to the removable nature of the device, which facilitates thorough cleaning.

The incorporation of the OHIP-14 instrument demonstrated that lingual brackets were also associated with heightened psychosocial and functional limitations. This finding may stem from speech difficulties, self-consciousness regarding breath odor, or discomfort. Our multivariate model reinforced these observations by isolating lingual bracket use as an independent driver of elevated HALT scores. Nevertheless, although patients with lingual brackets had a marginally longer treatment duration (0.4 months), adding this variable to the model left the appliance-type coefficients virtually unchanged. Notably, frequent snacking emerged as an aggravating factor, underscoring the nutritional dimension of halitosis management. Although the aligner group also included frequent snackers, their HALT and OHIP-14 scores remained comparatively lower, possibly due to the easier removal of aligners during meals and more efficient cleaning practices.

The correlation analysis indicated moderate to strong associations among HALT, organoleptic scores, and OHIP-14. Hence, halitosis should be recognized not only as a discrete condition but also as an important component of overall oral health-related quality of life. Future research may benefit from longitudinal designs that track changes in halitosis throughout the treatment cycle, along with more detailed dietary logs or instrumentation-based breath assessments (e.g., gas chromatography) to reduce subjective variability. From a clinical perspective, these findings highlight the need for orthodontists to address both mechanical and behavioral factors—appliance choice, rigorous hygiene protocols, and diet—in order to minimize halitosis and its social ramifications.

In examining the impact of different orthodontic treatments on oral health and quality of life, two studies provide detailed insights. Schaefer and Braumann’s research focused on patients undergoing Invisalign^®^ treatment, finding that the use of a low-dose chlorhexidine (CHX) solution significantly decreased volatile sulfur compounds, an indicator of halitosis, with their levels dropping to 0.06% during the first examination period. This study concluded that the adjunctive use of CHX might not be necessary with Invisalign^®^ due to minimal impairments in oral health-related quality of life [22]. Similarly, Jeong-Eun Kim et al. compared oral health outcomes between patients using fixed orthodontic appliances and those with clear aligners. The findings showed that patients with clear aligners had a gingival index score of 0.6, indicating mild gingivitis, compared to a score of 1.1 (moderate gingivitis) in the fixed appliance group [23]. Additionally, the clear aligner group had a lower degree of dental plaque deposition, with scores averaging 43.0, as opposed to 28.1 in the fixed appliance group. These studies highlight that patients with clear aligners not only manage to maintain better oral hygiene but do so with less frequent professional interventions like scaling and oral health education. Despite these differences in dental health metrics, patient satisfaction levels were similar across both groups, suggesting that both types of orthodontic treatments are well-received in terms of patient experience. These findings, along with our study results, reinforce the benefits of clear aligners for managing oral hygiene effectively, reflecting a shift towards less invasive orthodontic options in clinical settings.

The relationship between orthodontic treatments and halitosis has been substantively explored in recent studies, revealing nuanced insights into patient experiences and the effectiveness of different orthodontic appliances in managing oral malodor. In a study by Arwa A. Banjar et al. [24], it was found that 41.5% of participants perceived an increase in halitosis following the initiation of their orthodontic treatment, with the majority noticing this issue during and after the treatment process. This increase was significantly correlated with regular dental visits and a substantial rise in tongue coating formation among those with fixed appliances. Similarly, Jing Huang and colleagues conducted a systematic review and meta-analysis that also highlighted the link between fixed orthodontic appliances and exacerbated oral malodor [25]. Their findings demonstrated that malodor was notably higher after the first week of wearing fixed appliances. Interestingly, when different types of braces were compared, self-ligating brackets (SLBs) were more effective in controlling malodor than conventional brackets (CBs), with a mean difference of 4.32 in favor of SLBs. Both studies underscore that while orthodontic treatments are crucial for correcting malocclusions, they necessitate careful management of associated halitosis, with SLBs presenting a potential advantage over CBs in minimizing this undesirable side effect.

The impact of fixed orthodontic appliances on halitosis has been a focal point of recent orthodontic research, with studies exploring whether these devices contribute to increased oral malodor. In a systematic review by Salem Abdulraheem and colleagues [26], the evidence from three randomized clinical trials was evaluated to determine the effect of fixed orthodontic appliances on the development of halitosis. The review concluded that there is insufficient scientific evidence to definitively claim that fixed orthodontic appliances cause halitosis, citing the high risk of bias and very low quality of evidence in the available studies. In a similar manner, Oral Sökücü et al. [27] conducted a more focused study measuring oral malodor, the plaque index (PI), the gingival index (GI), and the probing pocket depth (PPD) over a year in orthodontic patients compared to controls. Their findings indicated that oral malodor significantly increased in the treatment group, with notable increases in the PI, GI, and PPD as well, particularly peaking at 7 months into treatment. These two studies present a contrasting view: while Abdulraheem et al. [26] call for more rigorous studies due to the current lack of conclusive evidence, Sökücü et al. [27] provide specific data showing a clear increase in malodor and associated oral health changes during orthodontic treatment. This juxtaposition highlights the need for high-quality, longitudinal studies to resolve these disparities and to refine orthodontic treatment protocols to mitigate potential negative effects like halitosis.

A principal limitation of this investigation is its cross-sectional nature, which restricts inferences regarding the causal direction between orthodontic appliance use and halitosis. Although the regression model helps parse out individual predictors, experimental or longitudinal evidence would offer stronger causality claims. Our sample, drawn from two university-affiliated clinics, may not reflect all demographics or private practice settings, limiting its generalizability. Additionally, self-report measures (snacking frequency, HALT, OHIP-14) are vulnerable to recall and social desirability biases. Organoleptic evaluations, while considered a standard in halitosis research, can be influenced by examiner subjectivity and environmental factors. Moreover, participants with extremely short or prolonged orthodontic treatments were not included, possibly underrepresenting certain complexities. Finally, due to resource constraints, we did not incorporate advanced breath analysis tools (e.g., volatile sulfur compound detectors), which might yield more precise quantitative malodor data. Future studies addressing these limitations could validate and expand upon our current findings.

## 5. Conclusions

In this cross-sectional analysis of removable aligner users, lingual bracket wearers, and non-orthodontic controls, multiple lines of evidence point to appliance design and dietary behaviors as critical factors in halitosis severity and oral health-related quality of life. Lingual brackets appear to pose the greatest challenge, with consistently higher HALT scores, elevated organoleptic findings, and more pronounced psychosocial impacts (as measured by OHIP-14). Frequent snacking further compounds these issues, likely by contributing to accelerated bacterial growth and plaque retention.

Removable aligners, conversely, exhibited a relative advantage in mitigating halitosis and preserving quality of life, suggesting that the ease of appliance removal and direct tooth cleaning may reduce microbial reservoirs. This practical benefit was evident regardless of snacking frequency, although minimizing frequent, sugary snacking remained beneficial in all groups. Taken together, these observations emphasize the importance of holistic patient education encompassing not only proper mechanical cleaning but also responsible dietary practices. Clinicians might adopt an individualized approach, tailoring hygiene protocols and nutritional guidance to the specific needs of lingual bracket wearers or aligner users. Additional prospective research is encouraged to evaluate how these findings evolve over time and to explore interventions that can further reduce halitosis-related burdens in orthodontic populations.

## Figures and Tables

**Table 1:** Demographic and clinical characteristics by orthodontic status.

Variable	Aligners (*n* = 55)	Lingual (*n* = 58)	Controls (*n* = 49)	*p*-Value
Age (years, mean ± SD)	22.7 ± 2.3	23.2 ± 2.6	22.4 ± 2.1	0.408
Female, *n* (%)	34 (61.8)	36 (62.1)	31 (63.3)	0.966 ^(1)^
Male, *n* (%)	21 (38.2)	22 (37.9)	18 (36.7)	-
Duration of ortho Tx (months, mean ± SD)	6.9 ± 1.6	7.3 ± 2.1	-	<0.001 ^(2)^
Frequent snacking, *n* (%)	24 (43.6)	30 (51.7)	18 (36.7)	0.128
Infrequent snacking, *n* (%)	31 (56.4)	28 (48.3)	31 (63.3)	-
Type of Snacking				0.072
Sugary snacks (primarily sweets, sweetened beverages), *n* (%)	29 (52.7)	35 (60.3)	19 (38.8)	
Non-sugary snacks (fruits, nuts, cheese, etc.), *n* (%)	26 (47.3)	23 (39.7)	30 (61.2)	
Brushing ≥ 2× /day, *n* (%)	45 (81.8)	46 (79.3)	33 (67.3)	0.108
Flossing ≥ 1× /day, *n* (%)	34 (61.8)	29 (50.0)	18 (36.7)	0.022
Mouthwash use ≥1 × /day, *n* (%)	28 (50.9)	35 (60.3)	17 (34.7)	0.018

^(1)^ Chi-square for female proportion across groups. ^(2)^ *p*-value pertains only to aligner vs. lingual bracket groups (no orthodontic duration for controls).

**Table 2:** HALT (Halitosis Associated Life-Quality Test) scores by group.

HALT Domain	Aligners (*n* = 55)	Lingual (*n* = 58)	Controls (*n* = 49)	*p*-Value (ANOVA)
Emotional Impact (0–25)	9.6 ± 2.2	11.3 ± 2.4	10.4 ± 2.5	0.005
Social Interactions (0–25)	8.4 ± 2.6	10.1 ± 2.7	9.1 ± 2.4	0.003
Personal Discomfort (0–25)	10.2 ± 2.9	11.9 ± 3.1	10.7 ± 2.8	0.016
Physical Concerns (0–25)	3.5 ± 1.8	4.1 ± 1.6	3.8 ± 1.9	0.131
Total HALT (0–100)	31.7 ± 5.8	37.4 ± 6.2	34.6 ± 6.0	<0.001

Data in the table presented as mean ± SD; Post hoc (Tukey) for Total HALT: aligners vs. lingual: *p* = 0.001; aligners vs. controls: *p* = 0.039; lingual vs. controls: *p* = 0.040.

**Table 3:** Organoleptic scores and snacking frequency.

Variable	Organoleptic Score (Mean ± SD)	*p*-Value
Frequent Snackers (*n* = 72)	2.3 ± 0.6	–
Infrequent Snackers (*n* = 90)	1.9 ± 0.5	<0.001 ^(1)^
Aligners (*n* = 55)	1.8 ± 0.5	–
Lingual (*n* = 58)	2.4 ± 0.6	<0.001 ^(2)^
Controls (*n* = 49)	2.0 ± 0.6	0.014 ^(3)^

^(1)^ Mann–Whitney test for frequent vs. infrequent snackers. ^(2)^ ANOVA comparison for lingual vs. aligners (*p* < 0.001). ^(3)^ Aligners vs. controls *p* = 0.049; lingual vs. controls *p* = 0.030 in Tukey post hoc.

**Table 4:** OHIP-14 (Oral Health Impact Profile) scores across groups.

OHIP-14 Domain	Aligners (*n* = 55)	Lingual (*n* = 58)	Controls (*n* = 49)	*p*-Value (ANOVA)
Functional Limitation (0–8)	1.5 ± 0.9	2.2 ± 1.1	1.9 ± 1.0	0.004
Physical Pain (0–8)	2.0 ± 1.2	2.5 ± 1.4	2.2 ± 1.3	0.096
Psychological Discomfort (0–8)	1.3 ± 1.0	2.0 ± 1.1	1.7 ± 1.0	0.009
Physical Disability (0–8)	1.1 ± 0.8	1.4 ± 1.0	1.2 ± 0.9	0.188
Psychological Disability (0–8)	1.2 ± 0.9	1.8 ± 1.1	1.4 ± 1.0	0.012
Social Disability (0–8)	1.4 ± 0.8	2.1 ± 1.0	1.5 ± 0.9	0.001
Handicap (0–8)	0.8 ± 0.7	1.3 ± 0.8	1.0 ± 0.7	0.005
Total OHIP-14 (0–56)	9.3 ± 3.5	12.3 ± 3.8	10.9 ± 3.7	<0.001

Data in the table presented as mean ± SD.

**Table 5:** Subgroup analyses: snacking frequency and total OHIP-14.

Snacking Frequency	Aligners (*n* = 55)	Lingual (*n* = 58)	Controls (*n* = 49)	*p*-Value
Frequent Snackers	(*n* = 24) 10.5 ± 3.3	(*n* = 30) 13.2 ± 3.7	(*n* = 18) 11.7 ± 3.5	0.018
Infrequent Snackers	(*n* = 31) 8.5 ± 3.1	(*n* = 28) 11.3 ± 3.4	(*n* = 31) 10.3 ± 3.8	0.021

Data in the table presented as mean ± SD; Overall ANOVA for each row: frequent snackers: *p* = 0.018; infrequent snackers: *p* = 0.021.

**Table 6:** Correlation matrix: HALT, organoleptic, OHIP-14, and snacking frequency.

Variables	HALT Total	Organoleptic	OHIP-14 Total	Snacking Frequency
HALT Total	1	+0.52	+0.59	+0.34
Organoleptic Score	+0.52	1	+0.45	+0.31
OHIP-14 Total	+0.59	+0.45	1	+0.28
Snacking Frequency	+0.34	+0.31	+0.28	1

Data in the table presented as mean ± SD.

**Table 7:** Multivariate regression: predictors of HALT score.

Predictor	β (Coefficient)	SE	*p*-Value
Lingual Appliance (ref: Controls)	3.1	1.2	0.011
Aligners (ref: Controls)	–2.2	1.1	0.046
Organoleptic Score	4.4	0.6	<0.001
OHIP-14 Total	0.6	0.1	<0.001
Frequent Snacking (ref: Infrequent)	2	0.9	0.029
Age (years)	0.1	0.1	0.112
Sex (Male = 1)	–0.7	1	0.494
Model Adjusted R^2^	0.42	-	-

## Data Availability

Data availability is subject to hospital approval.

## References

[1] Kapoor U., Sharma G., Juneja M., Nagpal A. (2016). Halitosis: Current concepts on etiology, diagnosis and management. Eur. J. Dent..

[2] Dadamio J., Van Tournout M., Teughels W., Dekeyser C., Coucke W., Quirynen M. (2013). Efficacy of different mouthrinse formulations in reducing oral malodour: A randomized clinical trial. J. Clin. Periodontol..

[3] Low B., Lee W., Seneviratne C.J., Samaranayake L.P., Hägg U. (2011). Ultrastructural changes associated with biofilm formation on metal and ceramic bracket materials. Scanning.

[4] Dawes C. (2008). Salivary flow patterns and the health of hard and soft oral tissues. J. Am. Dent. Assoc..

[5] Rosenberg M. (2002). The science of bad breath. Sci. Am..

[6] Bollen A.M. (2008). The effect of orthodontic therapy on periodontal health: A review of the literature. J. Orthod..

[7] Silva C.R., Silva C.C., Rodrigues R. (2022). Etiology of halitosis in pediatric dentistry. Arch. Pediatr..

[8] James A., Janakiram C., Meghana R.V., Kumar V.S., Sagarkar A.R. (2023). Impact of oral conditions on oral health-related quality of life among Indians—A systematic review and Meta-analysis. Health Qual. Life Outcomes.

[9] Rossini G., Parrini S., Castroflorio T., Deregibus A., Debernardi C. (2015). Periodontal health during clear aligners treatment: A systematic review. Eur. J. Orthod..

[10] Giannini L., Galbiati G., Tartaglia F.C., Grecolini M.E., Maspero C., Biagi R. (2025). Orthodontic Treatment with Fixed Appliances Versus Aligners: An Experimental Study of Periodontal Aspects. Dent. J..

[11] Mezied M.S., Al Bouri D., Al Omani A., Al Ramadhan G., Al Bootie S., Barakat A., Koppolu P. (2023). A Cross-Sectional Study on Self-Perceived Halitosis among Undergraduate University Students in Riyadh, Saudi Arabia. J. Pharm. Bioallied. Sci..

[12] Benson P.E., Dyer F., Macfarlane T.V. (2015). Relationships between dental appearance, self-esteem, and oral health-related quality of life in orthodontic patients. Eur. J. Orthod..

[13] Nalçacı R., Özat Y., Çokakoğlu S., Türkkahraman H., Önal S., Kaya S. (2014). Effect of bracket type on halitosis, periodontal status, and microbial colonization. Angle Orthod..

[14] Nazir M.A., Almas K., Majeed M.I. (2017). The prevalence of halitosis (oral malodor) and associated factors among dental students and interns, Lahore, Pakistan. Eur. J. Dent..

[15] Allaker R.P., Douglas C.W. (2009). Novel anti-microbial therapies for dental plaque-related diseases. Int. J. Antimicrob. Agents.

[16] Slade G.D. (1997). Derivation and validation of a short-form oral health impact profile. Community Dent. Oral Epidemiol..

[17] Papageorgiou S.N., Koletsi D., Iliadi A., Peltomäki T., Eliades T. (2020). Treatment outcome with orthodontic aligners and fixed appliances: A systematic review and meta-analysis. Eur. J. Orthod..

[18] Lalic M., Aleksic E., Gajic M., Milic J., Malesevic D. (2012). Does oral health counseling effectively improve oral hygiene of orthodontic patients?. Eur. J. Paediatr. Dent..

[19] Lommi S., Manzoor M., Engberg E., Agrawal N., Lakka T.A., Leinonen J., Kolho K.L., Viljakainen H. (2022). The Composition and Functional Capacities of Saliva Microbiota Differ Between Children with Low and High Sweet Treat Consumption. Front. Nutr..

[20] Patil P.S., Pujar P., Poornima S., Subbareddy V.V. (2014). Prevalence of oral malodour and its relationship with oral parameters in Indian children aged 7–15 years. Eur. Arch. Paediatr. Dent..

[21] Featherstone J.D.B., Crystal Y.O., Alston P., Chaffee B.W., Doméjean S., Rechmann P., Zhan L., Ramos-Gomez F. (2021). Evidence-Based Caries Management for All Ages-Practical Guidelines. Front. Oral Health.

[22] Schaefer I., Braumann B. (2010). Halitosis, oral health and quality of life during treatment with Invisalign^(®)^ and the effect of a low-dose chlorhexidine solution. J. Orofac. Orthop..

[23] Kim J.E., Kim S., Kim D.H. (2024). Comparison of oral health status, oral hygiene management behaviours and satisfaction of patients with fixed orthodontic appliance and clear aligner: A quasi-experimental design. Int. J. Dent. Hyg..

[24] Banjar A.A., Hassan S.M., Alyafi R.A., Alawady S.A., Alghamdi M.H., Baik K.M. (2022). Self-perceived halitosis among young adults undergoing orthodontic treatment. Int. J. Dent. Hyg..

[25] Huang J., Li C.Y., Jiang J.H. (2018). Effects of fixed orthodontic brackets on oral malodor: A systematic review and meta-analysis according to the preferred reporting items for systematic reviews and meta-analyses guidelines. Medicine.

[26] Abdulraheem S., Paulsson L., Petrén S., Sonesson M. (2019). Do fixed orthodontic appliances cause halitosis? A systematic review. BMC Oral Health.

[27] Sökücü O., Akpınar A., Özdemir H., Birlik M., Çalışır M. (2016). The effect of fixed appliances on oral malodor from beginning of treatment till 1 year. BMC Oral Health.

